# Comparative analysis of global and Chinese trends in the burden of ischemic stroke attributable to secondhand smoke from 1990–2021

**DOI:** 10.18332/tid/204510

**Published:** 2025-06-05

**Authors:** Xiao Zhou, Jiaming Liu, Xin Li

**Affiliations:** 1 Department of Neurology, The Second Hospital of Tianjin Medical University, Tianjin, China; 2 Tianjin Interdisciplinary Innovation Centre for Health and Meteorology, Tianjin, China

**Keywords:** GBD, ischemic stroke, secondhand smoke, BAPC, APC

## Abstract

**INTRODUCTION:**

Ischemic stroke (IS) represents a major health burden in China, and tobacco control is recognized as a practical and effective strategy to alleviate this impact. This study examines the influence of secondhand smoke (SHS) on the IS burden in China from 1990–2021.

**METHODS:**

Based on data from the Global Burden of Disease (GBD) 2021, this study analyzed the mortality and disability-adjusted life years (DALYs) burden of ischemic stroke (IS) attributable to secondhand smoke (SHS) in China and globally from 1990 to 2021. This study examined trends across different age and sex groups and projected future mortality and DALYs, providing a scientific basis for targeted public health strategies.

**RESULTS:**

Over the past 32 years, the number of IS deaths from SHS exposure in China rose from 23394.83 (95% UI: 15837.47–32315.19) in 1990 to 53697.88 (95% UI: 35003.84–76382.65) in 2021. Despite this increase, the ASMR declined from 3.94 (95% UI: 2.59–5.54) per 100000 population in 1990 to 2.92 (95% UI: 1.88–4.21) in 2021, with an AAPC of -0.04 (95% CI: -0.04–0.03). However, China's ASMR and ASDR remain significantly higher than the global average. APC analysis revealed greater mortality trends among the elderly and females. Over the next 30 years, mortality rates will decline across all ages, but deaths will rise, especially in those aged 75 years and older.

**CONCLUSIONS:**

The mortality rate of SHS-related IS in China declined from 1990 to 2021, but elderly and female patients still face a high burden. China’s disease burden remains higher than the global average. While mortality rates may continue to decline, deaths are expected to rise, especially among those aged 75 years and older.

## INTRODUCTION

Stroke is the second leading cause of death and the third leading cause of death and disability worldwide^1^. In 2019, there were 12.2 million new stroke cases, 101 million current stroke cases, and 6.55 million deaths from stroke worldwide. Compared with those in 1990, the number of stroke cases in 2019 increased by 70%, the number of patients increased by 85%, and the number of deaths increased by 43%^2^.

Ischemic stroke is characterized by a high incidence, high prevalence, high recurrence rate, high disability rate and high mortality rate^3^. Therefore, primary prevention plays a vital role in reducing the modifiable risk factors associated with IS^4^. Secondhand smoke (SHS) exposure, also known as passive or involuntary smoking, poses a major public health problem for non-smokers^5^. Approximately 37% of the world’s population is still exposed to tobacco products or exhaled smoke, and women and children have a greater exposure burden than men do, with significant racial and economic disparities^6-8^. Tobacco smoke contains thousands of chemicals and compounds, including many carcinogens, which pose health risks when inhaled^9^.

China is the largest tobacco consumer. According to the 2024 China Tobacco Industry Data Analysis Report, China is the country with the highest tobacco consumption, accounting for more than 43% of the world’s cigarette consumption^10^. Moreover, China is also a major cigarette producer^11^. Therefore, there is a gap in the comparative analysis between China and the global pattern, and there is a lack of longitudinal analysis of the IS burden. This study used 2021 GBD data, which cover 204 countries and 54 regions with different socioeconomic and demographic characteristics. Therefore, continuous monitoring and analysis of the IS burden and prevention and control work are needed to fully understand the epidemiological characteristics and risk factors for different countries and regions to promote more precise IS prevention and control strategies.

## METHODS

### Overview

The data were obtained from the GBD 2021 database (http://ghdx.healthdata.org/gbd-2021), which aims to assess the burden of 371 diseases and injuries across 21 global regions and 204 countries. All data were directly sourced from the GBD 2021 database, and the study did not involve medical ethics review or informed consent, and the Institutional Review Board (IRB) of Tianjin Medical University waived the requirement for informed consent, as only aggregated data were analyzed without involving any personally identifiable information. Similarly, the use of de-identified data from the GBD 2021 study was approved by the University of Washington’s IRB, which granted a waiver of informed consent. For this study, data were extracted by selecting the following parameters: GBD Estimate: Risk factor; Measure: Deaths and DALYs; Metric: Number and Rate; Risk: Secondhand Smoke; Cause: Ischemic Stroke. This study follows the guidelines outlined in the Strengthening the Reporting of Observational Studies in Epidemiology (STROBE) statement.

### Average annual percentage change

The Average Annual Percent Change (AAPC) and its 95% confidence intervals (CIs) are calculated by establishing an equation in which year is the independent variable and the incidence rate (expressed on a logarithmic scale) is the dependent variable. The AAPC comprehensively assesses the global average trend of changes over multiple time periods. Clegg et al.^[Bibr CIT0012]12^ proposed the Average Annual Percent Change (AAPC) model, which calculates the sum of slopes weighted by corresponding covariate subintervals. The assumptions and method selection for the AAPC model we used are based on this approach. We adopted the AAPC method proposed by Clegg et al.^12^ primarily due to its methodological characteristics: it directly calculates the sum of slopes weighted by subintervals, avoiding the arbitrary determination of trend inflection points. This method provides a clear and interpretable measure of annual change while accommodating potential fluctuations in the data^12^.

### Age-period-cohort model

The age-period-cohort (APC) model is a widely used statistical approach to uncover underlying patterns in mortality data, enabling the assessment of temporal influences such as age-specific risks, period-specific changes, and cohort-related factors. It helps disentangle the individual contributions of age, calendar year, and birth cohort to trends in stroke-related mortality. This model has been frequently applied in the epidemiological analysis of chronic diseases. In this context, the age effect captures the variation in risk among different age groups, while the period effect reflects temporal shifts in ischemic stroke (IS) mortality associated with secondhand smoke (SHS) that impact all age groups. The cohort effect considers the shared experiences or exposures of individuals born in the same period, especially early-life environmental and behavioral risk factors. The APC analysis in this study was conducted using the Analysis Tools provided by the National Cancer Institute (https://analysistools.nci.nih.gov/apc/help.html).

### Bayesian age-period-cohort models with projections

The Bayesian Age-Period-Cohort Model (BAPC) is a statistical model used to analyze and predict the impact of age, time period, and birth cohort on an event (such as mortality, disease incidence, etc.) in population data. The BAPC model combines the advantages of Bayesian statistical methods and can handle complex data structures and uncertainties (https://folk.ntnu.no/andrerie/software.html). In this study, the R software package (version 4.2.3), and JD_GBDR (V2.22, Jingding Medical Technology Co., Ltd.) and GraphPad Prism 10 were used for plotting.

## RESULTS

### The burden of SHS-related IS in China from 1990 to 2021 and comparison with global levels

Comprehensive data of SHS-related IS deaths, DALYs, and their respective age-standardized rates in China from 1990 to 2021 are given in Table 1. The total number of deaths due to SHS-related IS increased from 23394.83 (95% UI: 15837.47– 32315.19) in 1990 to 53697.88 (95% UI: 35003.84–76382.65) in 2021, representing a 129.5% growth. The age-standardized mortality rate (ASMR) per 100000 population decreased from 3.94 (95% UI: 2.59–5.54) to 2.92 (95% UI: 1.88–4.21), with an AAPC of -0.04 (95% CI: -0.04 – -0.03). The trend of DALYs change is similar to that of death, with an AAPC of -0.71 (95% CI: -0.77 – -0.66). The results in Supplementary file Figure 1 show that the mortality rate and DALYs of SHS-related IS in women in China are significantly higher than those in men.

**Table 1:** All-age cases and age-standardized rates of mortality and DALYs, by gender, from ischemic stroke attributable to secondhand smoke exposure in China and globally in 1990 and 2021

*Location*	*1990*		*2021*		*AAPC (95% CI)*
	*n (95% UI)*	*ASR per 100000* *population* *(95% UI)*	*n (95% UI)*	*ASR per 100000* *population* *(95% UI)*	
**Deaths**					
**Global**	88678.46 (60524.16–121138.38)	2.67 (1.80–3.68)	123790.74 (81620.64–168952.06)	1.50 (0.99–2.06)	-0.038 (-0.039 – -0.036)
Male	30552.11 (20803.28–42390.16)	2.19 (1.46–3.05)	54094.81 (35148.91–73857.02)	1.52 (0.99–2.09)	-0.022 (-0.023 – -0.020)
Female	58126.35 (39160.02–79291.96)	2.99 (2.01–4.09)	69695.94 (46266.21–96329.90)	1.49 (0.99–2.06)	-0.048 (-0.050 – -0.046)
**China**	23394.83 (15837.47–32315.19)	3.94 (2.59–5.54)	53697.88 (35003.84–76382.65)	2.92 (1.88–4.21)	-0.036 (-0.040 – -0.033)
Male	8025.27 (5147.01–11777.11)	3.33 (2.10–4.87)	24349.95 (14964.59–35297.82)	3.20 (1.93–4.63)	-0.006 (-0.010 – -0.003)
Female	15369.56 (10187.74–21463.70)	4.49 (2.90–6.35)	29347.93 (18583.04–43471.15)	2.80 (1.76–4.18)	-0.054 (-0.060 – -0.048)
**DALYs**					
**Global**	2011605.19 (1385147.47–2714948.44)	53.51 (36.87–72.46)	2741835.15 (1819494.99–3680991.52)	32.26 (21.30–43.39)	-0.685 (-0.714 – -0.656)
Male	197865.20 (128813.64–286916.42)	60.06 (39.32–86.05)	508468.24 (312207.94–739470.71)	56.66 (34.81–81.72)	-0.400 (-0.427 – -0.372)
Female	391325.11 (263322.32–539768.38)	96.21 (63.99–133.24)	657183.56 (430090.60–938270.73)	61.26 (40.07–88.04)	-0.903 (-0.933 – -0.874)
**China**	589190.32 (403803.74–804039.26)	78.44 (52.87–108.41)	1165651.80 (774274.81–1600441.50)	58.26 (38.22–80.24)	-0.714 (-0.771 – -0.657)
Male	732153.02 (503867.79–1008489.68)	43.56 (29.90–60.55)	1213455.59 (791382.06–1633068.39)	31.27 (20.25–42.10)	-0.119 (-0.188 – -0.049)
Female	1279452.16 (874631.04–1711029.69)	61.38 (42.17–82.39)	1528379.55 (1028459.82–2088766.82)	33.26 (22.44–45.43)	-1.126 (-1.216 – -1.036)

From 1990 to 2021, the ASMR and ASDR of SHS-related IS in China were significantly higher than the global average (Supplementary file Figure 1). The ASMR and ASDR of SHS-related IS in China and the world have both decreased, whereas the corresponding numbers of deaths and DALYs have increased (Table 1; and Supplementary file Figure 1).

### Gender-based variations in the SHS-related IS burden across age groups in China

**Figure 1:**
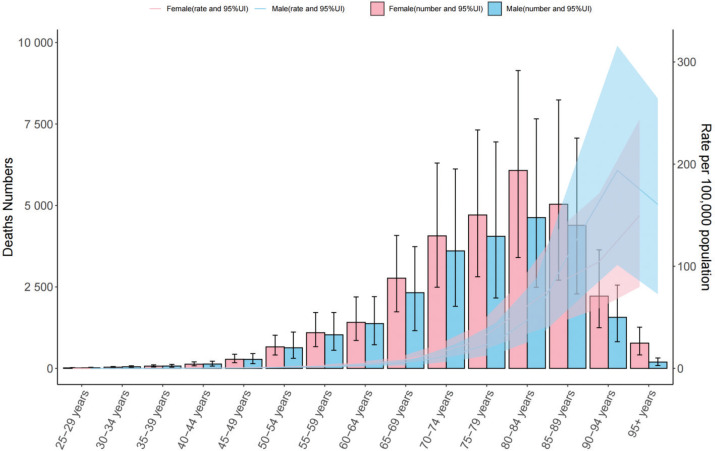
Gender and age differences in ischemic stroke attributable to secondhand smoke in China, 2021

Figures 1 and Supplementary file Figures 2 and 3 show that in 2021, the age-standardized mortality rate (ASMR) of men and women in all age groups in China and globally tended to first increase but then decrease with age and peaked in the 80–84 years age group. With increasing age, the mortality gap between men and women gradually widened, especially in the elderly group. In addition, among people aged 90 years and above, the mortality rate of men tended to decrease. In Supplementary file Figure 2 can be seen that the trend of the age-standardized disability-adjusted life-year rate (ASDR), which is similar to the overall trend of the ASMR, but its peak appeared earlier, reaching the highest value in the 70–74 years age group.

Compared with 1990 (Supplementary file Figure 3), the gender difference in SHS-related IS has changed significantly. In 1990, the burden of SHS-related IS disease in women was significantly higher than that in men, but by 2021, this gap had narrowed. In addition, the age distribution of the disease burden has also changed. The peak age of SHS-related IS burden in 2021 was delayed compared with that in 1990, reflecting that the disease burden is more concentrated in the older population.

### APC effects on SHS-related IS mortality

**Figure 2:**
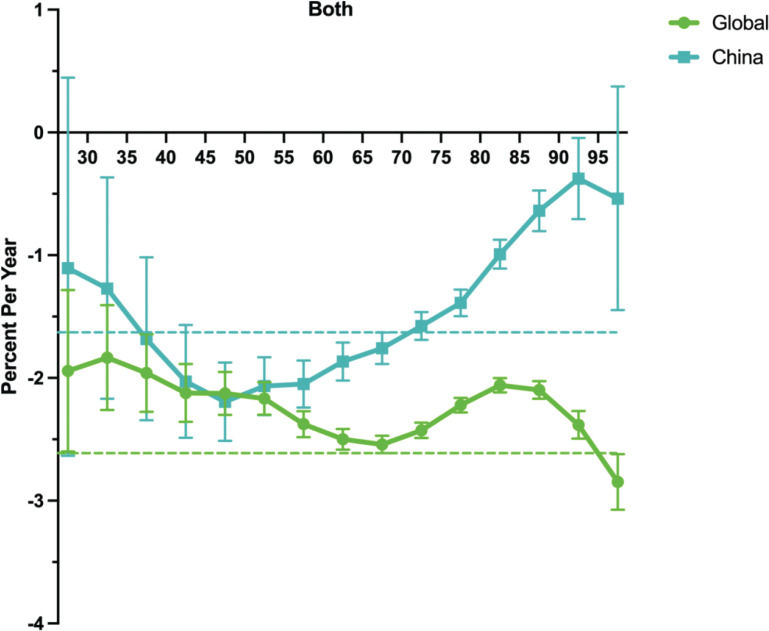
Cohort trends in ischemic stroke mortality attributable to secondhand smoke, with corresponding 95% confidence intervals: China versus Global, 1992–2021

The net drift represents the average annual percentage change over the entire study period, whereas the local drift indicates the annual percentage change in mortality rate for each age group relative to the net drift. The analysis revealed divergent trends in secondhand smoke (SHS)-attributable stroke mortality between China and the global average. While both showed declining mortality rates, China’s annual net drift (-1.58%, 95% CI: -1.72 – -1.44) demonstrated a significantly slower reduction compared to the global trend (-2.26%, 95% CI: -2.33 – -2.19) (Figure 2). Local drift reflects additional age variations in mortality trends. Across all age groups, the values for both males and females in China and globally were below 0, indicating an improvement in mortality rates. However, the trends differed. In China, the local drift rate showed a downward trend among the 30–50 years age group, reaching its lowest point around the age of 50 years, before starting to rise. It then increased sharply in the elderly population, particularly among those aged 85 years and above. In contrast, the global local drift rate exhibited a relatively stable downward trend across most age groups, with an increase observed in individuals aged 90 years and above, although this increase was less pronounced than that in China. Supplementary file Figure 4B illustrates the local drift trend in females. In China, the mortality rate change remained relatively stable before the age of 50 years and then gradually declined, followed by a sharp increase after the age of 70 years. Globally, the local drift rate in females showed an overall downward trend, but the magnitude of change was smaller than that in China. Supplementary file Figure 4A presents the local drift trend in males. In China, the local drift rate among males declined in the 30–50 years age group, reaching its lowest point at approximately 50 years of age, and then gradually increased, with a significant surge among those aged 85 years and above. In contrast, the global male local drift rate remained relatively stable across most age groups, showing a slight decline, followed by an increase in those aged 80 years and above, although to a less extent than in China.

Overall, the local drift rate in China exhibited a more substantial decline among younger individuals (30–50 years), whereas in the elderly population (80 years and older), it rose significantly (Supplementary file Figure 4C). The longitudinal age distribution revealed that SHS-related IS mortality initially increased but then decreased with age. A similar pattern was observed for the cohort effect (Supplementary file Figure 4D). Compared with the global trend, the incidence rate among the elderly in China has increased more significantly, particularly in those aged 80 years and above, whose risk is much higher than the global average. With respect to the period effect, we observed a continuous decline in SHS-related IS mortality, with a more pronounced decrease globally (Supplementary file Figure 4E).

### Projected trends in SHS-related IS mortality for the next 30 years

**Figure 3:**
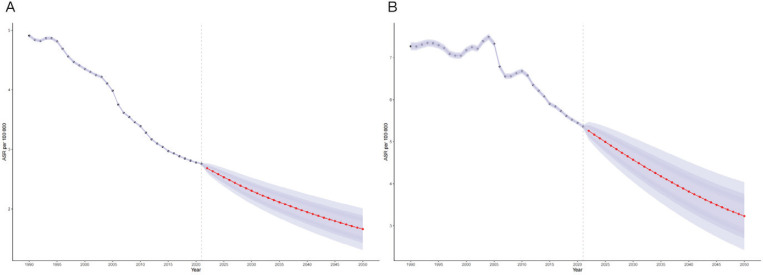
Future projections of mortality due to ischemic stroke attributable to secondhand smoke: A) Trend of age specific rate globally; B) Trend of age specific rate in China. The open dots represent the observed values, and the fan shape, the predicted distribution between the 50 and 95% quantiles. The average forecast is shown as a solid line. The vertical dotted line indicates where the prediction begins

The results from the Bayesian Age-Period-Cohort (BAPC) prediction model indicate that SHS-related IS mortality is expected to decline in all age groups from 2022 to 2050 (Figure 3; and Supplementary file Figure 5). The magnitude of the decline varies across age groups, with a more rapid decline in younger groups and a more gradual change in older groups. Despite the decline in age-standardized mortality rates, the absolute number of deaths due to ischemic stroke is expected to increase significantly, especially in the 75 years and older age group, due to the growing aging population. This trend is also evident worldwide (Supplementary file Figure 6). Furthermore, gender-stratified analyses showed trends consistent with both the overall patterns observed in China and those seen globally (Supplementary file Figures 7 and 8).

## DISCUSSION

On the basis of the latest GBD 2021 data, this study systematically analyzed the disease burden of SHS-related IS in China over the past 30 years and compared the ASMR and ASDR of SHS-related IS in China from 1990 to 2021 with those at the global level. The results revealed that the ASMR and ASDR of SHS-related IS in China were significantly higher than the global average, and the decline in these two indicators in China was also lower than the overall global trend. Although the burden rate of SHS-related IS in China has been declining, the absolute number of related deaths and DALYs continues to rise due to factors such as population aging and changes in the total population, indicating that the public health impact of SHS on the stroke burden is still severe^13^. Our analysis further emphasized that the disease burden of SHS-related IS is closely related to the age of patients and generally is associated with increased mortality in middle-aged and elderly individuals. This finding is consistent with existing research results^14^. With increasing age, the risk of the coexistence of many chronic diseases, such as cardiovascular disease, obesity, type II diabetes, dyslipidemia, non-alcoholic fatty liver disease, anxiety, depression, and inflammatory bowel disease^15-17^. These comorbidities are more common in the middle-aged and elderly population, which may further aggravate the disease burden of SHS-related IS.

Decades of research have confirmed that there is a close link between tobacco use and cerebrovascular disease. Currently, smoking and passive smoking are considered independent risk factors for ischemic stroke^18^. A study of the Chinese population revealed that passive smoking increased the overall risk of stroke in the general population by 45% and the risk of death after stroke by 2-fold^19^. The pathogenesis of SHS-related IS involves several factors: harmful components such as nicotine and carbon monoxide in tobacco can directly damage vascular endothelial cells and destroy their structure and function^20,21^, thereby inducing inflammatory responses, promoting low-density lipoprotein (LDL) oxidation, and ultimately accelerating the process of atherosclerosis. In addition, endothelial dysfunction can reduce the bioavailability of nitric oxide (NO)^22^, weaken vasodilation, and further increase the risk of ischemic stroke^23^. Smoking can increase platelet adhesion and aggregation and promote the activation of coagulation factors, thereby significantly increasing the risk of thrombosis^24^. Nicotine can activate the sympathetic nervous system, leading to increased catecholamine levels, which in turn cause increased heart rate and blood pressure. Long-term hypertension can cause continuous mechanical stress on the vascular wall, promote vascular remodeling and hardening, and increase susceptibility to stroke^25^. Smoking can induce contraction of vascular smooth muscle cells, leading to spasm of small arteries, thereby increasing peripheral vascular resistance and blood flow shear force. Long-term and repeated vasoconstriction not only aggravates endothelial damage but also may induce local ischemia or thrombosis, further increasing the risk of ischemic stroke^26^.

Although the mortality and DALY rates of SHS-related IS in China have been steadily declining from 1990 to 2021, the absolute burden has continued to rise and is higher than the global average. There are 316 million smokers in China, and approximately 740 million non-smokers are exposed to secondhand smoke, with more than 100000 deaths each year^27^. In addition, the public is exposed to secondhand smoke in a wide range of places. The results of the 2018 China Adult Tobacco Survey revealed that restaurants had the highest secondhand smoke exposure rate of 73.3%, followed by families at 50.9%, workplaces at 44.9%, and government buildings at 31.1%^28^. In contrast, residents in wealthy areas of high-income countries have a higher level of education and a greater degree of participation in tobacco control and are generally less exposed to SHS^29^. Children in low- and middle-income countries suffer more frequent IS attacks because of greater SHS exposure^30^. Globally, approximately 19% of men and 33% of women are exposed to secondhand smoke (SHS), with women facing a greater risk. In our study, women consistently exhibited a higher burden of SHS-related ischemic stroke compared to men. This disparity may be partly explained by sociocultural factors. In many settings, women are more likely to experience secondhand smoke exposure within the household environment due to traditional gender roles and lower smoking prevalence among women themselves. Occupational differences, such as employment in sectors with limited smoking restrictions, may also contribute to elevated SHS exposure among women. These factors may help explain the observed gender differences in the burden of SHS-related ischemic stroke. Therefore, comprehensive smoke-free policies have more significant health benefits for people in areas with low and medium-low social development indices (SDIs), especially women. The disease burden of ischemic stroke (IS) caused by SHS exposure in these areas showed a more obvious downward trend after the implementation of smoke-free policies. Although the current smoke-free policy has achieved positive results, its importance cannot be ignored. This further highlights the need for continuous optimization and innovation of policy making to promote broader and deeper tobacco control measures to minimize SHS-related health risks.

At present, 181 countries have signed the WHO Framework Convention on Tobacco Control and adopted a series of tobacco control measures. Ireland passed smoke-free legislation in March 2004 and became the first country to have smoke-free public places^31^, followed by Spain, New Zealand, Norway and 12 other countries. Studies have shown that within one year after the Irish law came into effect, the secondhand smoke exposure level of non-smoking bar staff decreased, and the salivary cotinine level decreased by 80%^32^. Within one year after the implementation of national smoke-free legislation in England, the salivary cotinine level of non-smoking adults decreased by 80%^33^. Five years after Italy implemented a comprehensive smoking ban, the exposure rate of secondhand smoke in public places was 10.2%. The Italian smoking ban has greatly reduced exposure to secondhand smoke in public places^34^. According to statistics, 107 cities in China currently have enacted special smoke-free legislation for public places. Since the signing of the Framework Convention on Tobacco Control in 2006, a total of 70 cities have enacted special legislation on smoking in public places or revised tobacco control legal documents. However, most smoke-free legislation is concentrated in economically developed areas. In underdeveloped areas, exposure to secondhand smoke in public places is still relatively common, and the coverage and enforcement of tobacco control policies are relatively insufficient. Such regional disparities may lead to increased health inequalities, with people in economically underdeveloped areas, especially women, children, and low-income people, continuing to face increased risks of secondhand smoke exposure. In addition, although some cities have established smoke-free regulations, challenges remain in implementation and supervision, such as uneven law enforcement, low public awareness and compliance with smoke-free policies, and continued high exposure to secondhand smoke in private places (such as homes and private vehicles).

### Implications

To further reduce SHS-related health hazards, China should learn from successful international experiences and strengthen the coverage of smoke-free policies. First, the enforcement of smoke-free legislation should be strengthened, and unified enforcement standards and supervision mechanisms should be established to ensure that smoke-free policies are effectively implemented. Second, continuous public publicity and education should be carried out through multiple channels to increase public awareness and support for the hazards of secondhand smoke and smoke-free policies, especially in rural and underdeveloped areas. Third, efforts should be made to strengthen protection for vulnerable groups, and special tobacco control intervention strategies should be formulated for women, children and low-income groups to promote health equity.

### Limitations

This study has several limitations. First, the accuracy and comprehensiveness of GBD statistics largely rely on the quality and availability of data, including disease diagnosis and measurement of secondhand smoke (SHS) exposure levels. In particular, in areas with limited medical resources and people with low socioeconomic status, the diagnosis of IS may be imperfect, and the assessment of secondhand smoke exposure may be insufficient. This situation is particularly evident in areas with low and medium sociodemographic indices (SDIs) worldwide. In addition, self-reported secondhand smoke exposure may deviate from objective measurements, which may lead to underestimation or overestimation of the relevant disease burden. Second, inconsistencies in disease classification and coding may introduce potential bias and affect the reliability of the research results. Moreover, with the advancement of diagnostic technology and changes in data collection methods, the comparability of data across different time periods may be affected. In addition, the disease burden assessment provided by GBD 2021 data is limited mainly to the national and regional levels, so this study cannot conduct a more detailed analysis of the SHS-related IS burden in various provinces in China. This study used the BAPC model to predict future mortality rates, and there is a certain discrepancy between the projected population and the actual population.

## CONCLUSIONS

This study still reveals the significant disease burden of SHS-related IS in China, emphasizes the importance of continuous monitoring of epidemic trends, and explores the impact of socio-economic development on the disease burden. These insights are highly important for formulating more effective public health policies, raising social awareness of the hazards of SHS, and taking precise prevention and control measures for different populations.

## Supplementary Material



## Data Availability

The data supporting this research are available from https://vizhub.healthdata.org/gbd-results/
