# Supplementary material for: Comparative analysis of global and Chinese trends in the burden of ischemic stroke attributable to secondhand smoke from 1990–2021
Source: Tob Induc Dis. 2025 Jun 5;23:10.18332/tid/204510. doi: 10.18332/tid/204510 (PMC12139392; doi:10.18332/tid/204510)
Supplement: Supplementary file 1 [file TID-23-78-s1.pdf]

SUPPLEMENTAL MATERIAL

eMethods 1. Data Collection
eMethods 2. Abbreviation
eMethods 3. STROBE Statement—checklist of items that should be included in reports of observational studies
Figure 1. The burden of ischemic stroke attributable to secondhand smoke in China and globally in 1990 to 2021 :（A）ASMR;（B）The number of mortality;（C）ASDR; (D)The number of DALYs.
Figure 2. Ischemic stroke attributable to secondhand smoke in China and globally gender age differences in 2021.(A,B)China; (C,D) Global
Figure 3. Ischemic stroke attributable to secondhand smoke in China and globally gender age differences in 1990.(A,B)China; (C,D) Global
Figure 4. Parameter estimates of age, period, and cohort effects on Ischemic stroke attributable to secondhand smoke mortality rate in China and global from 1990 to 2021. Local drift with net drift values for IS mortality (A) Male (B) Female in China and Global from 1990 to 2021.C, Fitted longitudinal age curves of IS mortality (per 100 000 person-years) and the corresponding 95% CIs. D, Relative risk of each period for age and nonlinear cohort effects and the corresponding 95% CI. E, Relative risk of each cohort for age and nonlinear period effects and the corresponding 95% CI (some were too narrow to show).
Figure 5. Future projections of mortality due to ischemic stroke attributable to secondhand smoke.(A) Trend of Age specific cases globally; (B) Trend of Age specific cases in China
Figure 6. Future projections of mortality due to ischemic stroke attributable to secondhand smoke.(A) Trend of Age specific rate globally; (B) Trend of Age specific rate in China; (C) Trend of Age specific cases globally; (D) Trend of Age specific cases in China
Figure 7. Future projections of mortality among men due to ischemic stroke attributable to secondhand smoke.(A) Trend of Age specific rate globally; (B) Trend of Age specific rate in China; (C) Trend of Age specific cases globally; (D) Trend of Age specific cases in China
Figure 8. Future projections of mortality among female due to ischemic stroke attributable to secondhand smoke.(A) Trend of Age specific rate globally; (B) Trend of Age specific rate in China; (C) Trend of Age specific cases globally; (D) Trend of Age specific cases in China

eMethods 1. Data Collection

The data used in this study comes from the Global Burden of Disease Study 2021 (GBD 2021). This study provides comprehensive data on incidence, prevalence, mortality, and Disability-Adjusted Life Years (DALYs) by country, time, age, and gender. It is the most comprehensive global epidemiological observational study to date. GBD 2021 data is collected from multiple sources, including censuses, household surveys, civil registration and vital statistics, disease registries, healthcare utilization, air pollution monitoring, satellite imagery, disease reporting, and others. These data sources are uniquely identified in the Global Health Data Exchange (GHDx) and provide metadata as well as data shared by providers with permission for dissemination. Researchers can explore the data sources used for evaluating the burden of EHCE diseases worldwide via the GBD 2021 Data Input Sources Tool available at https://vizhub.healthdata.org/gbd-results/.

After correcting for known biases, the GBD network uses the Bayesian meta-regression tool, DisMod-MR 2.1, for analysis. This tool comprehensively evaluates the available data on incidence, prevalence, and mortality for each disease, ensuring consistency among various epidemiological parameters to produce estimates of disease burden. The model can generate estimates for countries lacking original data, thereby enabling global evaluation of the EHCE burden across all countries. The methodology employed by GBD 2021, including data input and processing, is detailed in previous research literature. The statistical data provided by GBD 2021 includes estimates and their 95% uncertainty intervals, which are derived from the 25th and 975th percentiles of 1,000 estimates based on the GBD algorithm. All statistical data is reported per 100,000 population.

eMethods 2. Abbreviation

Abbreviation

Abbreviation	Full name
APC	Age-period-cohort
IS	Ischemic Stroke
SHS	Secondhand Smoke
GBD	Global Burden of Disease
DALYs	Disability-adjusted Life Years
AAPC	Average annual percent change
ASMR	Age-standardized Mortality Rate
ASDR	Age-standardized DALY Rate
IHME	Institute for Health Metrics and Evaluation
BAPC	Bayesian age-period-cohort
LDL	Low-density lipoprotein
NO	Nitric Oxide
SDIs	Social development indices
UI	Uncertainty Interval
CI	Confidence Interval

eMethods 3. STROBE Statement—checklist of items that should be included in reports of observational studies

STROBE Statement—checklist of items that should be included in reports of observational studies

	Item No	Recommendation
**Title and abstract**	1	(*a*) Indicate the study's design with a commonly used term in the title or the abstract
		(*b*) Provide in the abstract an informative and balanced summary of what was done and what was found
**Introduction**		
Background/rationale	2	Explain the scientific background and rationale for the investigation being reported
Objectives	3	State specific objectives, including any prespecified hypotheses
**Methods**		
Study design	4	Present key elements of study design early in the paper
Setting	5	Describe the setting, locations, and relevant dates, including periods of recruitment, exposure, follow-up, and data collection
Participants	6	(*a*) *Cohort study*—Give the eligibility criteria, and the sources and methods of selection of participants. Describe methods of follow-up<br>*Case-control study*—Give the eligibility criteria, and the sources and methods of case ascertainment and control selection. Give the rationale for the choice of cases and controls<br>*Cross-sectional study*—Give the eligibility criteria, and the sources and methods of selection of participants
		(*b*) *Cohort study*—For matched studies, give matching criteria and number of exposed and unexposed<br>*Case-control study*—For matched studies, give matching criteria and the number of controls per case
Variables	7	Clearly define all outcomes, exposures, predictors, potential confounders, and effect modifiers. Give diagnostic criteria, if applicable
Data sources/ measurement	8*	For each variable of interest, give sources of data and details of methods of assessment (measurement). Describe comparability of assessment methods if there is more than one group
Bias	9	Describe any efforts to address potential sources of bias

Study size	10	Explain how the study size was arrived at
Quantitative variables	11	Explain how quantitative variables were handled in the analyses. If applicable, describe which groupings were chosen and why
Statistical methods	12	(*a*) Describe all statistical methods, including those used to control for confounding
		(*b*) Describe any methods used to examine subgroups and interactions
		(*c*) Explain how missing data were addressed
		(*d*) *Cohort study*—If applicable, explain how loss to follow-up was addressed<br>*Case-control study*—If applicable, explain how matching of cases and controls was addressed<br>*Cross-sectional study*—If applicable, describe analytical methods taking account of sampling strategy
		(*e*) Describe any sensitivity analyses

Continued on next page

**Results**		
Participants	13*	(a) Report numbers of individuals at each stage of study—eg numbers potentially eligible, examined for eligibility, confirmed eligible, included in the study, completing follow-up, and analysed
		(b) Give reasons for non-participation at each stage
		(c) Consider use of a flow diagram
Descriptive data	14*	(a) Give characteristics of study participants (eg demographic, clinical, social) and information on exposures and potential confounders
		(b) Indicate number of participants with missing data for each variable of interest
		(c) *Cohort study*—Summarise follow-up time (eg, average and total amount)
Outcome data	15*	*Cohort study*—Report numbers of outcome events or summary measures over time
		Case-control study—Report numbers in each exposure category, or summary measures of exposure
		Cross-sectional study—Report numbers of outcome events or summary measures
Main results	16	(*a*) Give unadjusted estimates and, if applicable, confounder-adjusted estimates and their precision (eg, 95% confidence interval). Make clear which confounders were adjusted for and why they were included
		(*b*) Report category boundaries when continuous variables were categorized
		(*c*) If relevant, consider translating estimates of relative risk into absolute risk for a meaningful time period
Other analyses	17	Report other analyses done—eg analyses of subgroups and interactions, and sensitivity analyses
**Discussion**		
Key results	18	Summarise key results with reference to study objectives
Limitations	19	Discuss limitations of the study, taking into account sources of potential bias or imprecision. Discuss both direction and ma gnitude of any potential bias
Interpretation	20	Give a cautious overall interpretation of results considering objectives, limitations, multiplicity of analyses, results from similar studies, and other relevant evidence
Generalisability	21	Discuss the generalisability (external validity) of the study results
**Other information**		
Funding	22	Give the source of funding and the role of the funders for the present study and, if applicable, for the original study on which the present article is based

*Give information separately for cases and controls in case-control studies and, if applicable, for exposed and unexposed groups in cohort and cross-sectional studies.

Note: An Explanation and Elaboration article discusses each checklist item and gives methodological background and published examples of transparent reporting. The STROBE checklist is best used in conjunction with this article (freely available on the Web sites of PLoS Medicine at http:// www.plosmedicine.org/, Annals of Internal Medicine at http://www.annals.org/, and Epidemiology at http://www.epidem.com/). Information on the STROBE Initiative is available at www.strobe-statement.org.

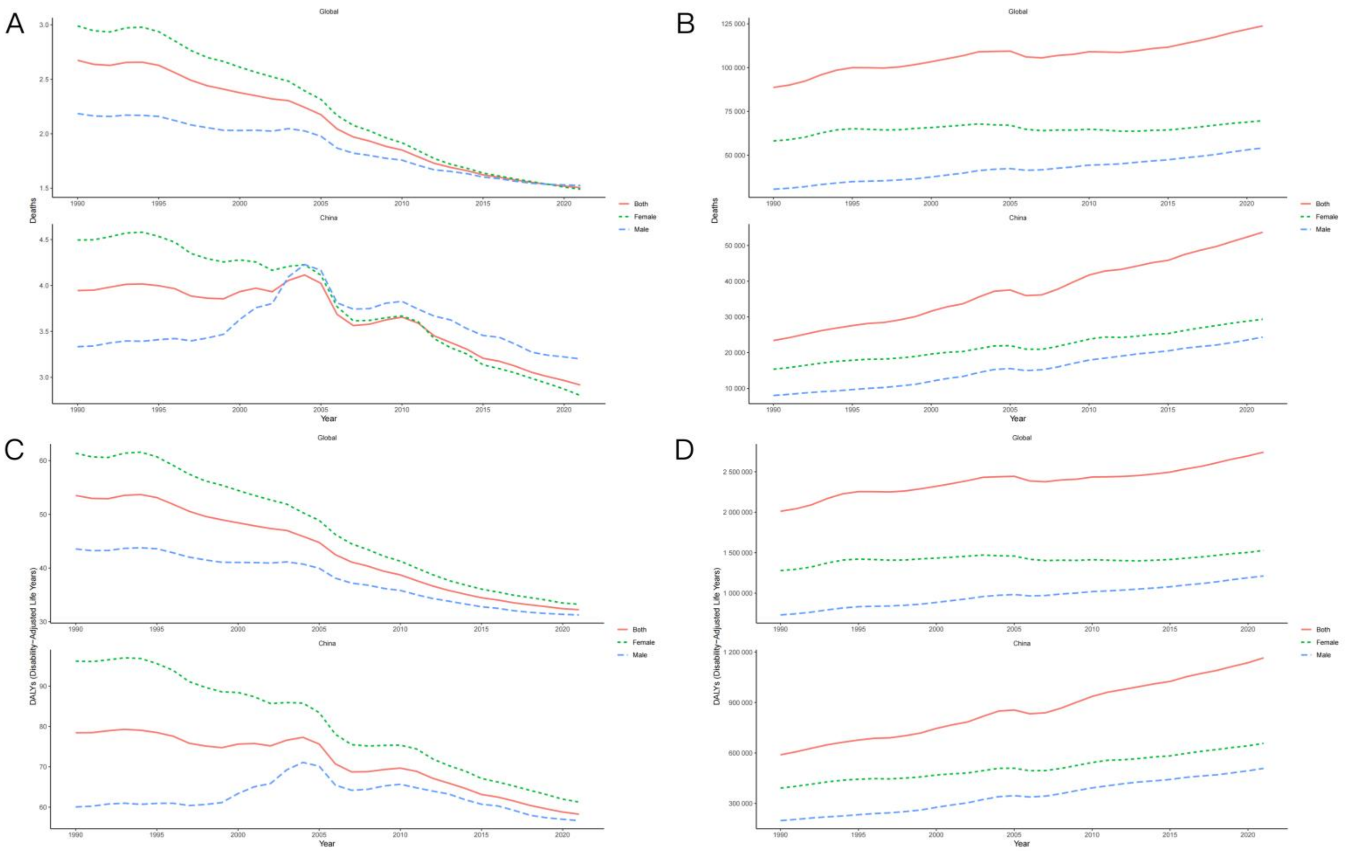

Figure 1. The burden of ischemic stroke attributable to secondhand smoke in China and globally in 1990 to 2021 :（A）ASMR;（B）The number of mortality;（C）ASDR; (D)The number of DALYs.

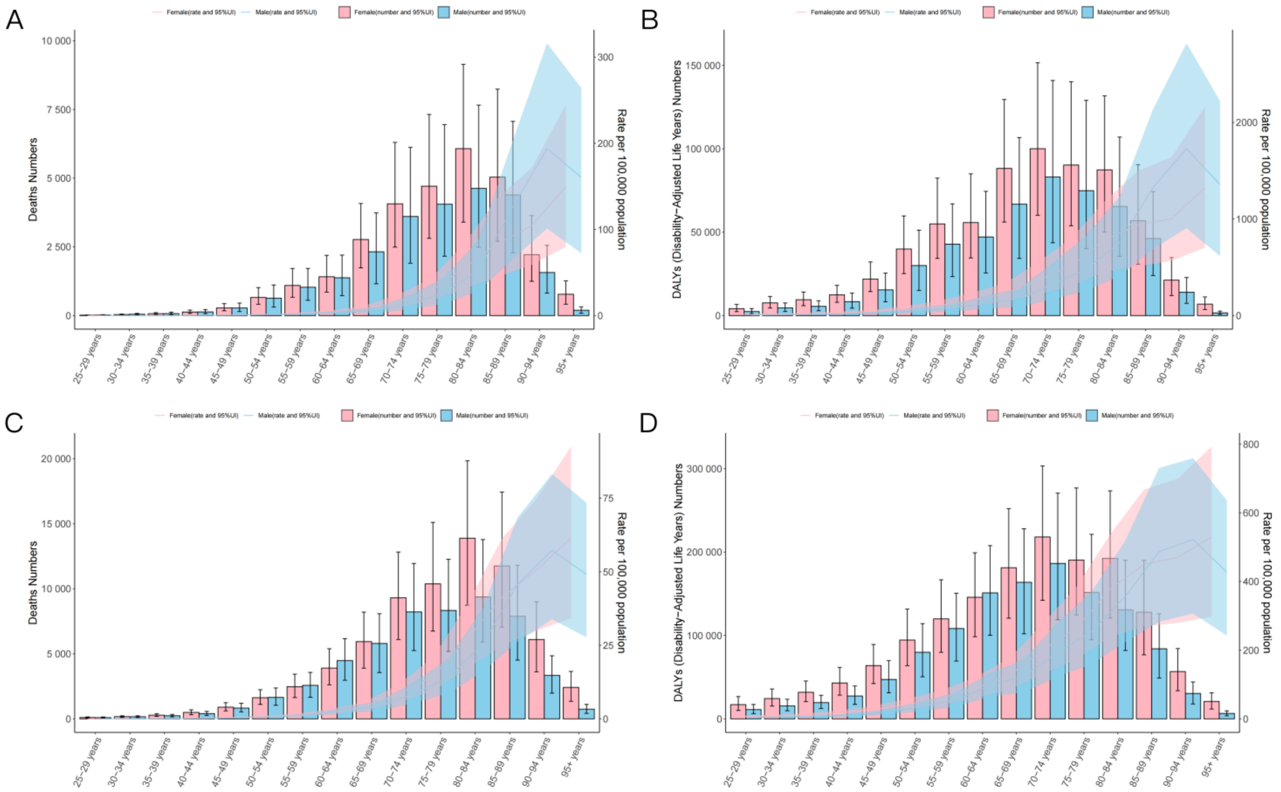

Figure 2. Ischemic stroke attributable to secondhand smoke in China and globally gender age differences in 2021.(A,B)China; (C,D) Global

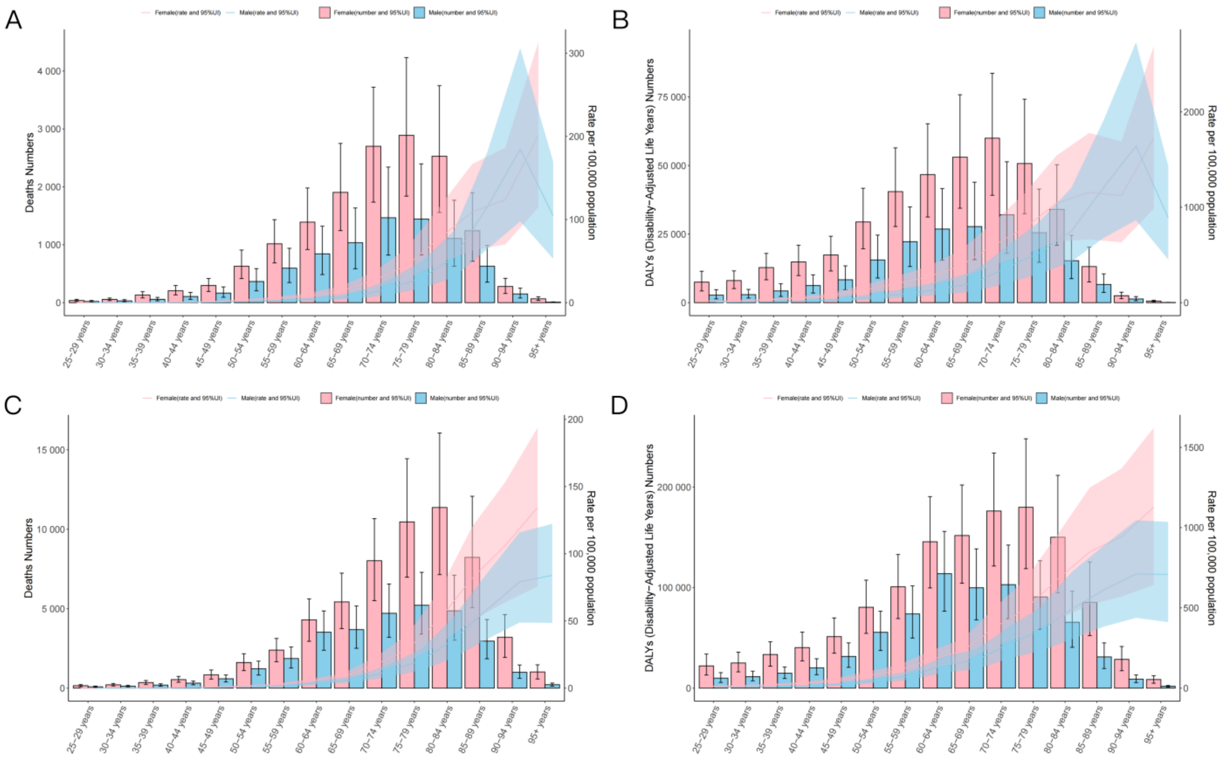

Figure 3. Ischemic stroke attributable to secondhand smoke in China and globally gender age differences in 1990.(A,B)China; (C,D) Global

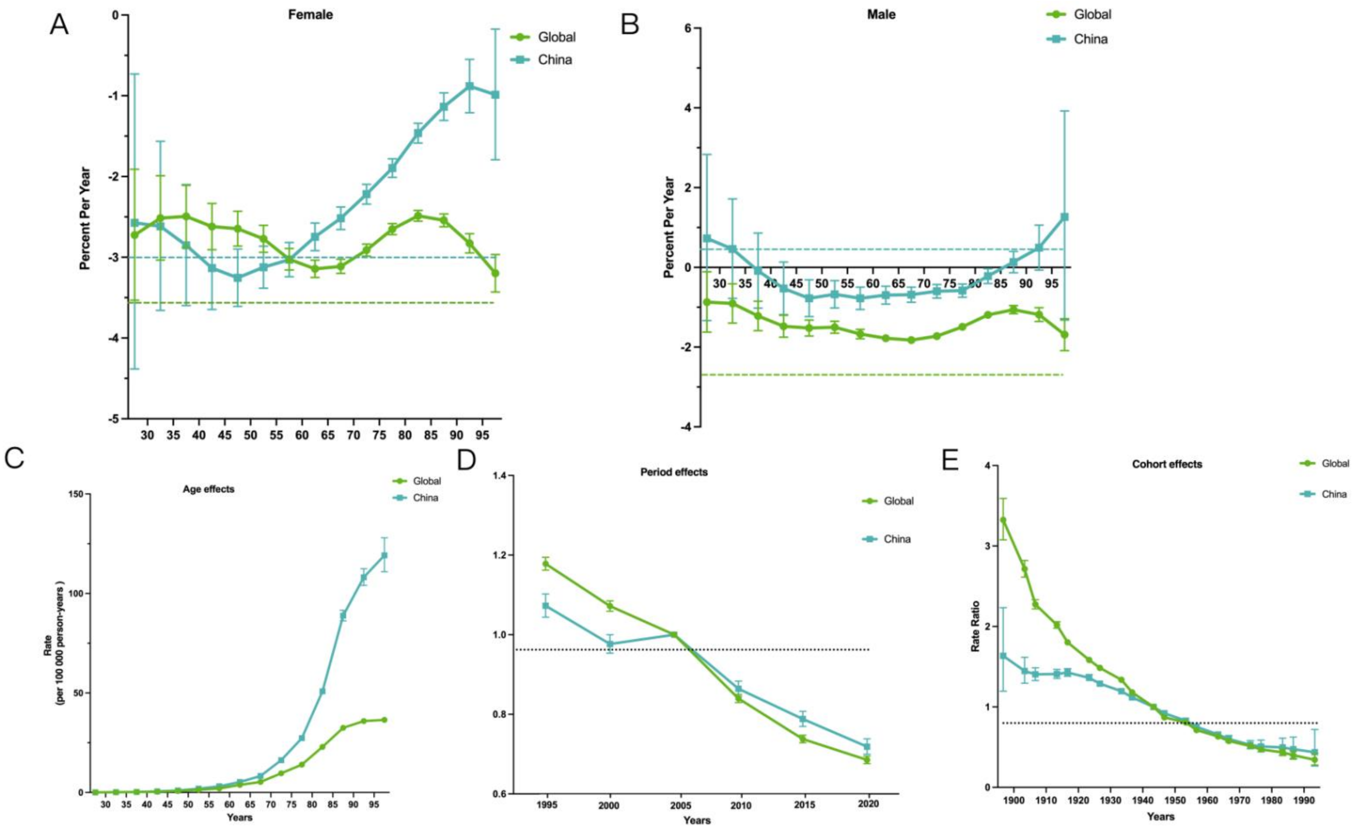

Figure 4. Parameter estimates of age, period, and cohort effects on Ischemic stroke attributable to secondhand smoke mortality rate in China and global from 1990 to 2021. Local drift with net drift values for IS mortality (A) Male (B) Female in China and Global from 1990 to 2021.C, Fitted longitudinal age curves of IS mortality (per 100 000 person-years) and the corresponding 95% CIs. D, Relative risk of each period for age and nonlinear cohort effects and the corresponding 95% CI. E, Relative risk of each cohort for age and nonlinear period effects and the corresponding 95% CI (some were too narrow to show).

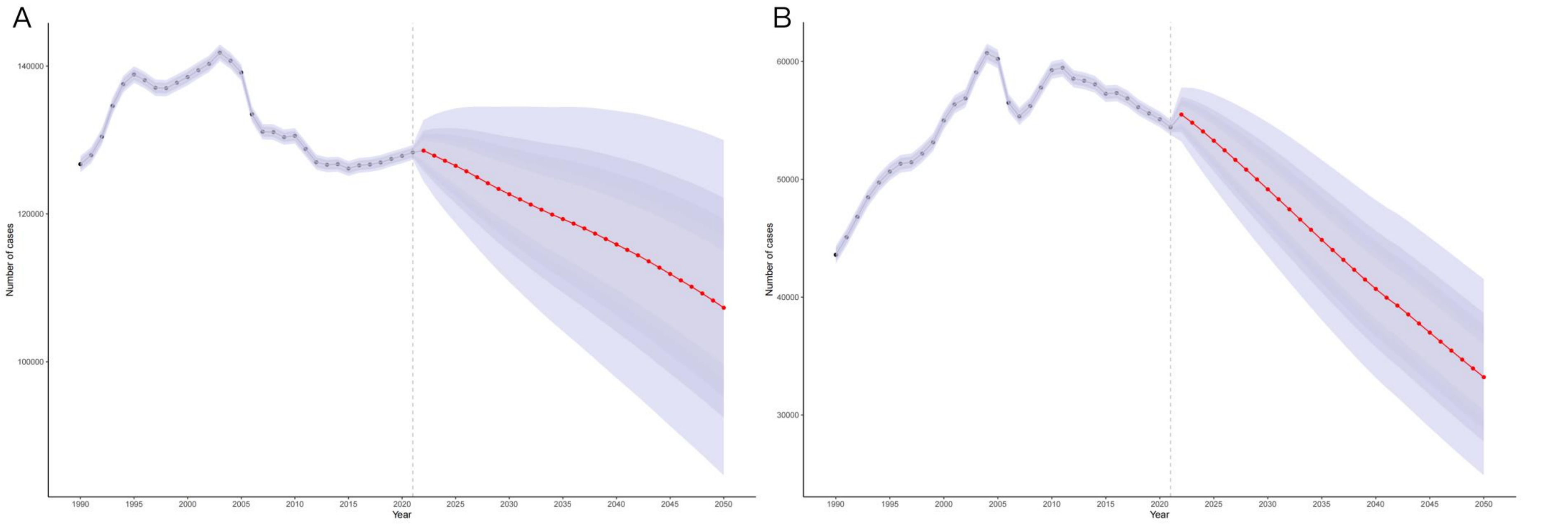

Figure 5. Future projections of mortality due to ischemic stroke attributable to secondhand smoke.(A) Trend of Age specific cases globally; (B) Trend of Age specific cases in China

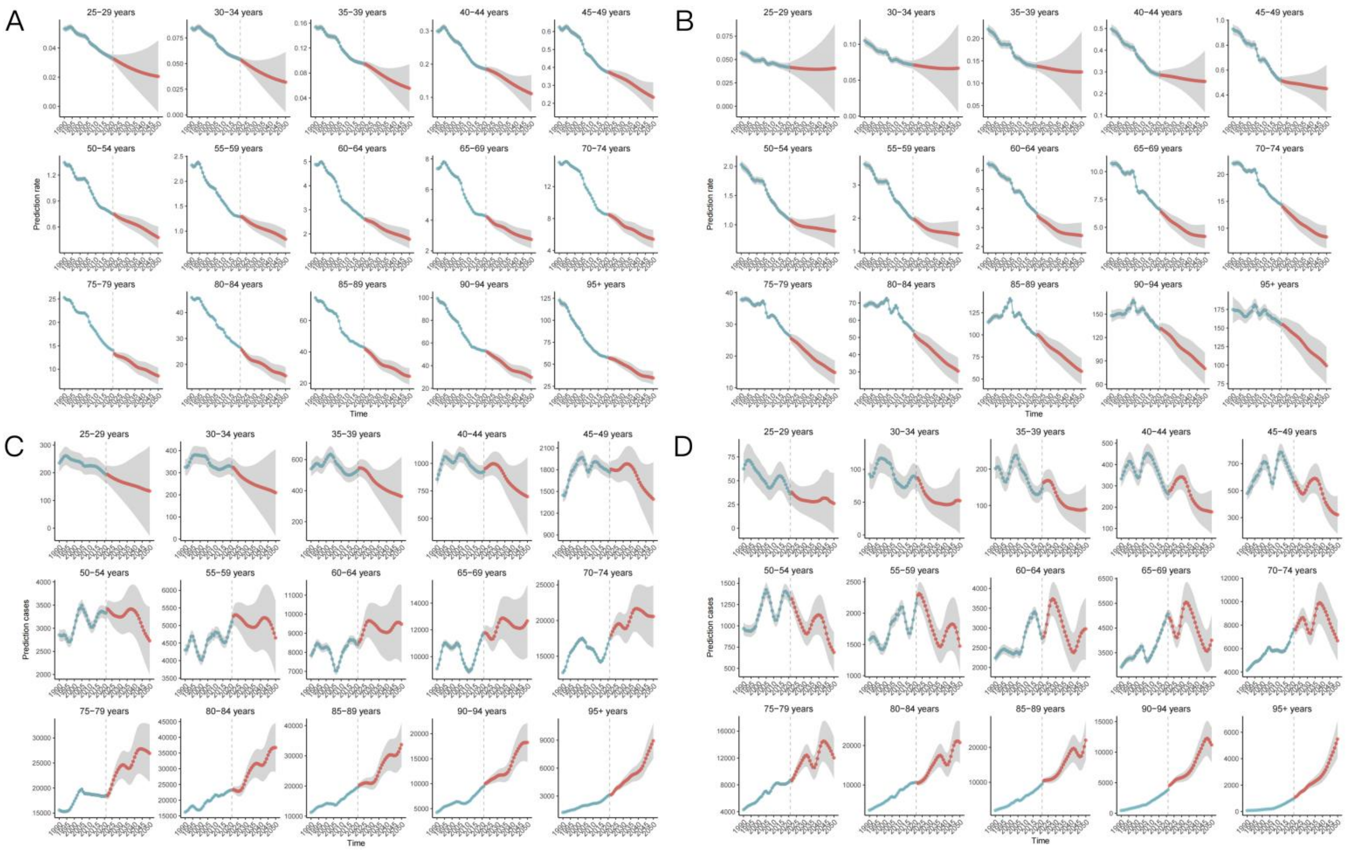

Figure 6. Future projections of mortality due to ischemic stroke attributable to secondhand smoke.(A) Trend of Age specific rate globally; (B) Trend of Age specific rate in China; (C) Trend of Age specific cases globally; (D) Trend of Age specific cases in China

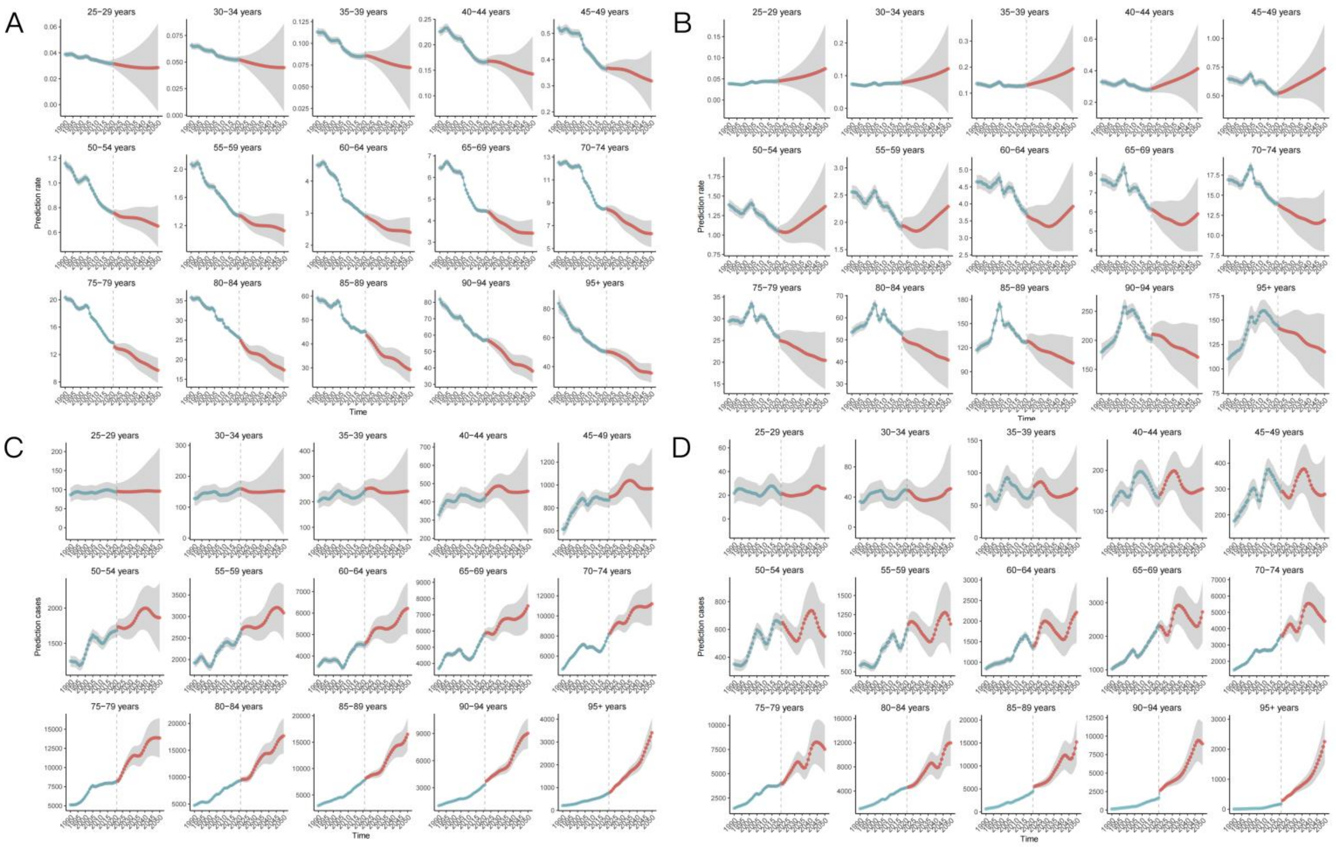

Figure 7. Future projections of mortality among men due to ischemic stroke attributable to secondhand smoke.(A) Trend of Age specific rate globally; (B) Trend of Age specific rate in China; (C) Trend of Age specific cases globally; (D) Trend of Age specific cases in China

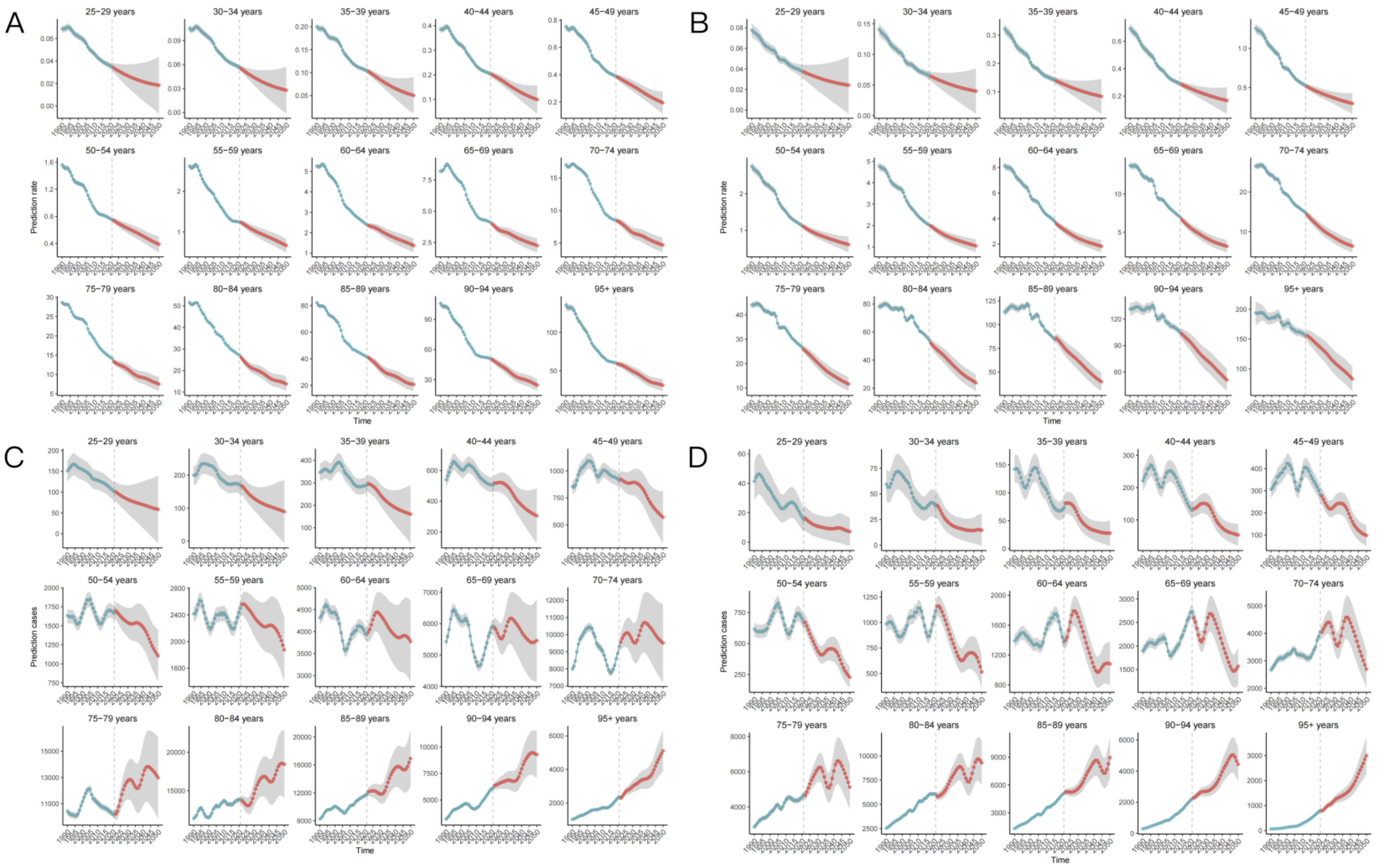

Figure 8. Future projections of mortality among female due to ischemic stroke attributable to secondhand smoke.(A) Trend of Age specific rate globally; (B) Trend of Age specific rate in China; (C) Trend of Age specific cases globally; (D) Trend of Age specific cases in China

© 2025 Zhou X. et al.
